# Viscount Burnham and Maitre De Leval on Nurse Cavell

**Published:** 1919-05-24

**Authors:** 


### VISCOUNT BURNHAM AND MAITRE DE LEVAL ON NURSE CAVELL.

### Her Last Words Recorded.

On the day following the funeral scenes in London and Norwich Maître Gaston de Leval, the distinguished Belgian who did his best to save the life of Nurse Cavell, told to a large gathering in the Queen's Hall, London, the full story of her arrest, trial and death, introducing new facts and disclosing names which during the war could not be mentioned. The meeting was held under the auspices of the Edith Cavell Homes of Rest, for which movement, during the afternoon, a collection with appeals were made for financial support.

Viscount Burnham, who presided, gave the assurance that the monument, by Sir George Frampton, to the heroic memory of Edith Cavell, which was to be erected in the heart of London, would be worthy of its purpose and its subject. Edith Cavell, he said, had the nurse's trained spirit of devotion to those who suffer, and she went to her death with a fortitude and dignity that will stand as the pattern of English conduct. The previous day she had the burial of a queen among women; the whole people did reverence to the radiance of her self-sacrifice. (Applause.) The noble Chairman, in concluding, asked help for the Edith Cavell Homes of Rest movement, which was collecting funds to equip places where nurses can find restoration of health and reinvigoration of spirit—places which it was the heart's desire of Edith Cavell to see scattered throughout the land. The need is even greater than was anticipated.

### Secret History Revealed.

Maître de Leval said that from what he had seen during the past two days he realised that in England Nurse Cavell was really a national heroine. In every British heart there was love for the woman who had done more for the success of the war in dying than many generals had done in living. The whole life of London City was stopped to see a poor woman, a feeble woman, without any rank, passing to her last abode; the woman the Germans had so despised that they told him "The smallest German soldier is worth more to us than all your English nurses." In that funeral the soul of a people had revealed itself. It was veneration for what Nurse Cavell stood for—a symbol of sacrifice and duty to the last.

Dealing with events before and after the arrest of Nurse Cavell, Maître de Leval testified that it was the Prince de Croy, who lived with his sister in a château near Mons, who took the leading part in the work of smuggling refugee soldiers over the Dutch frontier. Nurse Cavell, M. Severin and M. Baucq organised a system by which these soldiers, sent to Brussels by the Prince and his sister, were concealed in the nursing home. Two other women assisted in the work—the Countess de Belleville and Mdlle. Thuilliez. Owing to a spy thirty-seven persons were arrested; Prince de Croy escaped. Nurse Cavell told the truth from the start. She had saved men from death. Was not that her usual task? She acted in the cause of humanity; she did not dream of any political end. The prosecutor at the secret trial was a person named Stoeber, for whom we could not have too much hatred. He had lost a son in the war, and was furious with the English. He bullied Nurse Cavell in a most horrible way. He told her she lied, treated her like a slave, and asked for the death sentence, which was passed, though under the strict code it could not apply to Nurse Cavell's offence. The sentence was kept secret from all Nurse Cavell's friends, in spite of their efforts to learn the truth. Advised by one of her fellow prisoners to make a plea for pardon, Nurse Cavell replied, with a smile, "No, I am English—they want my life." And they got it. Some people said that at the execution she fainted. Those who said that did not know Nurse Cavell. She was not a woman to faint. Five minutes before dying she wrote these lines on her Prayer Book, "Arrested August 5, sentenced to death October 11, died October 12, seven in the morning. With love to my mother."

In concluding, Maître de Leval said: In the funeral ceremony at Westminster some people were missing. I would have brought Stoeber there; I would have brought Von Saubersweig; I would have brought the Kaiser. (Loud applause.) I would have made them kneel down on those stones on which English soldiers were marching, carrying the body of Nurse Cavell. (A Voice: They should be shot.) Justice will come; if it was for me to decide they would be shot. Than Nurse Cavell there is in history no better example of an admirable woman. Never shall we be able to think enough of her courage and her virtues. (Loud applause.)

### French Legion of Honour.

The Comte André d'Ormesson, from the French Embassy, then presented to Mrs. Wainwright (Nurse Cavell's sister) the Legion of Honour.—Dr. Wainwright expressed the thanks of the family for this tribute from France.

Sir Richard C. Temple, Bart. (Chairman of the Edith Cavell Homes of Rest for Nurses), in proposing a vote of thanks, said the Homes were started to accommodate 120 nurses a year. That was soon found insufficient. They could now accommodate between 600 and 700 a year, but that covered hardly half the need. The other day they had 100 women, worn out by the war and needing help, waiting, but they had no room for them.

Maître de Leval, replying, said there were going to be many statues to Nurse Cavell, but homes were more useful than statues, and he commended this movement to the public.

### A Brussels Memorial.

The British Minister at Brussels announces that an influential Committee has been formed to promote the erection of a worthy monument in Brussels to Nurse Edith Cavell. The King of the Belgians has consented to be Patron, and the Committee includes the names of Mr. Lloyd George, Mr. Balfour, Lord Curzon, Mr. Asquith, the Earl of Athlone, Mr. Delacroix, Prime Minister of Belgium, and others. The erection of a statue, the further development of the nursing institutions in which Miss Cavell was interested, and the foundation of an International Hospital in Brussels are some of the aims under discussion. Subscriptions may be sent to Messrs. Hoare, 37 Fleet Street, E.C. 4, marked "Edith Cavell Memorial Fund."

